# Supplementary material for: Evidence for compositionally distinct upper mantle plumelets since the early history of the Tristan-Gough hotspot
Source: Nat Commun. 2023 Jul 3;14:3908. doi: 10.1038/s41467-023-39585-0 (PMC10318034; doi:10.1038/s41467-023-39585-0)
Supplement: Supplementary file 1 — Supplementary Information [file 41467_2023_39585_MOESM1_ESM.pdf]

Supplementary figures

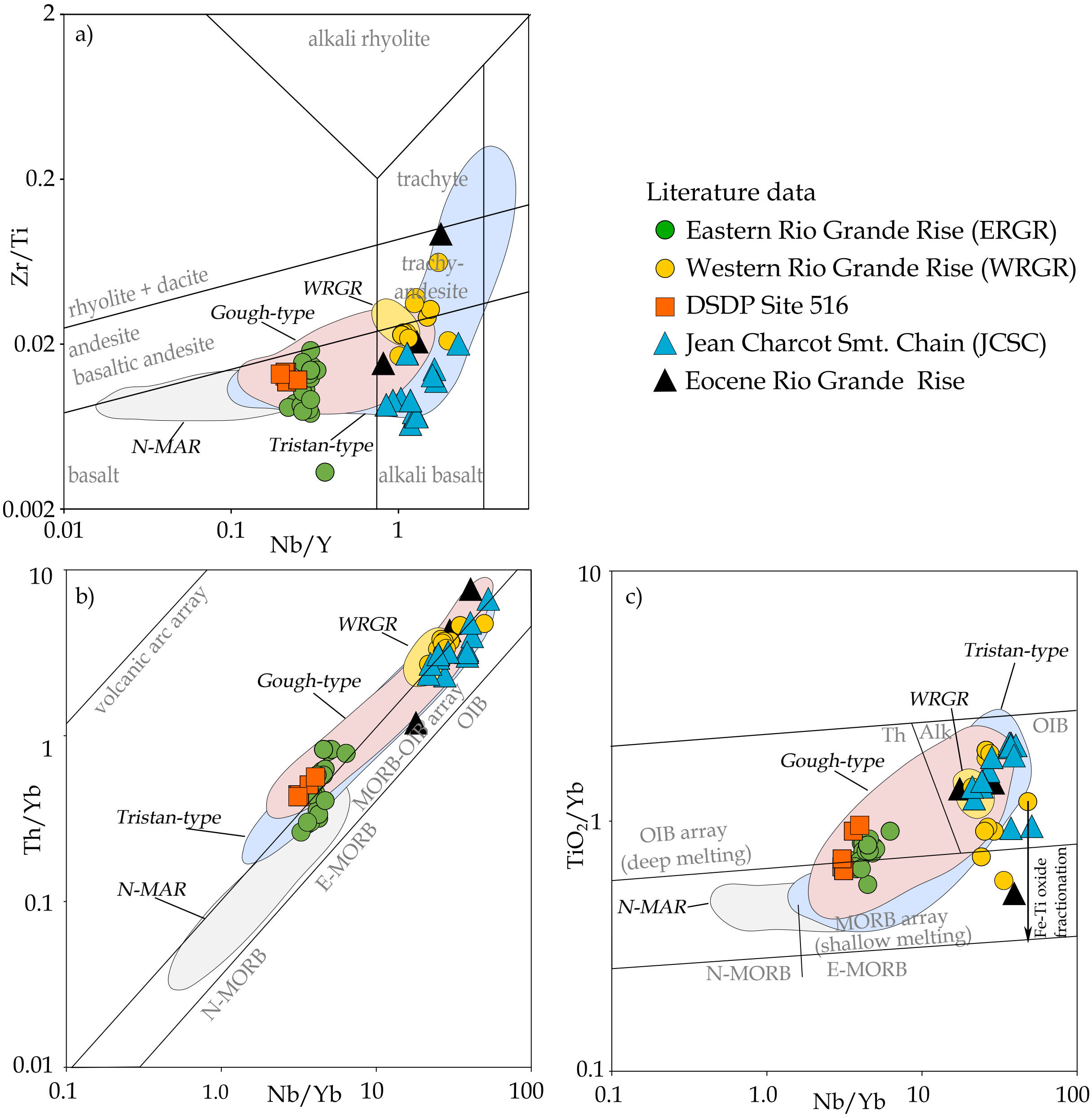

Fig. S1: Immobile incompatible trace element discrimination diagrams from Pearce [1] and Pearce [2]. a) Nb/Y vs. Zr/Ti, b) Nb/Yb vs. Th/Yb and c) Nb/Yb vs. TiO_2/Yb of the JCSC and RGR samples. For comparison, fields for the two distinct (Tristan and Gough) EMI compositional types from the Tristan-Gough seamount chain and North Atlantic Mid-Ocean Ridge basalts (N-MAR) are shown. References for literature data are given in Fig. 2 caption. On Nb/Yb vs Th/Yb discrimination diagram (Pearce [2], the tholeiitic rocks lie within the E-MORB compositional range, whereas the alkaline rocks have OIB-like compositions (**Fig. S1b**). All samples fall within the Tristan and Gough compositional range, which straddle the upper mantle array boundary[2].

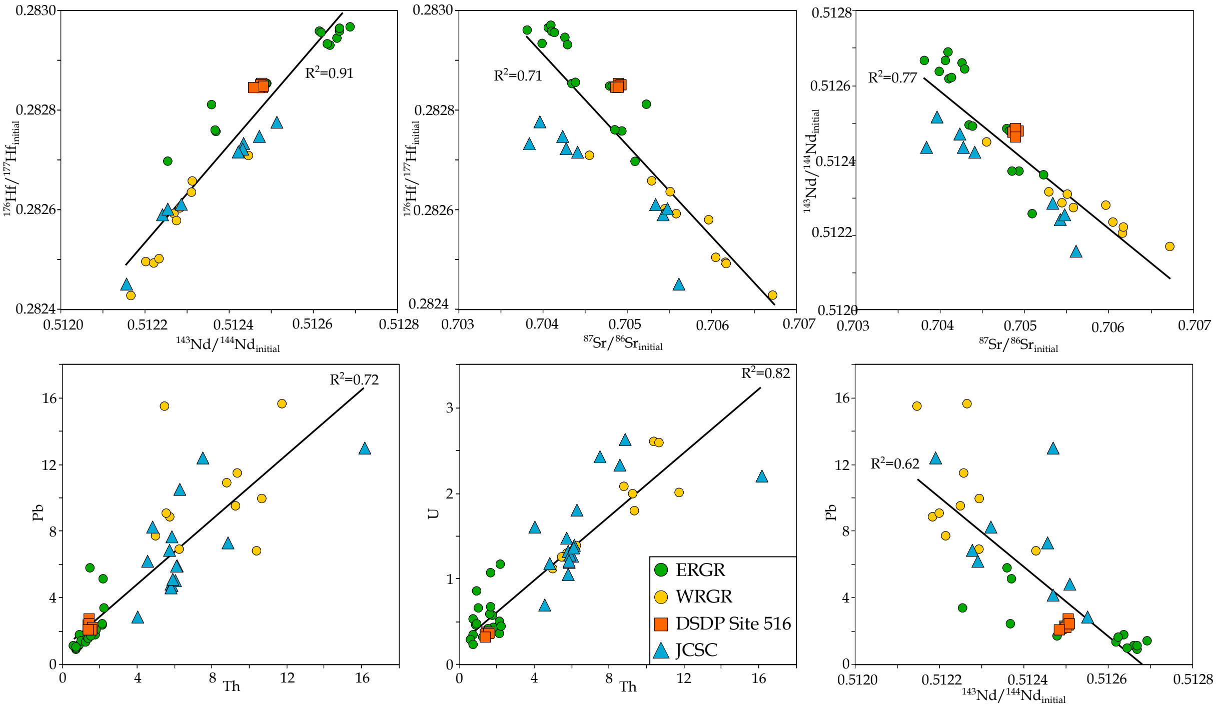

Fig. S2: The good correlation between mobile and immobile trace elements and radiogenic isotopes indicates that the studied rocks are not significantly affected by alteration. Additionally, the regression lines and best fit are shown.

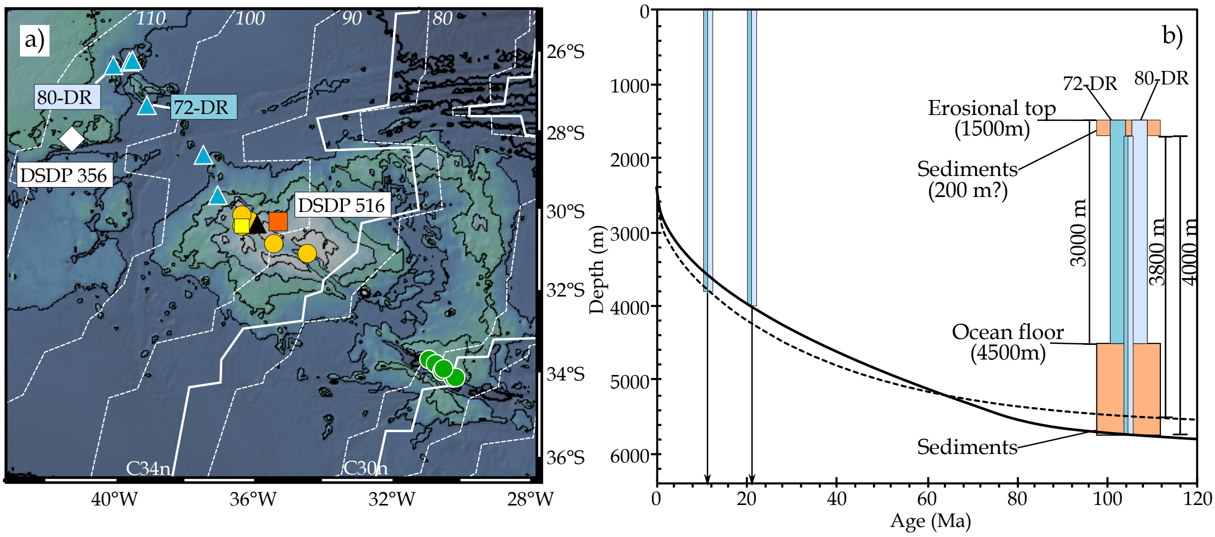

Fig. S3: Bathymetric map and age estimate of the northernmost Jean-Charcot Seamounts. a) shows the locations and b) our age estimates of dredge sites 72-DR and 80-DR on guyot-like structures in the northernmost JCSC[3], which rise approximately 3000 m from the ocean floor at ~4500 mbsl (meter below sea level) to the flat top/plateau at ~1500 mbsl. The discrepancy between the depth of the seafloor and the magmatic top of the ocean crust is related to a thick sediment cover[4, 5]. Seismic data close to 72-DR indicates a sediment thickness of ~1500 m[6], whereas DSDP Site 356 recovered a 700 m thick sediment column emplaced over 100 Ma[7]. Therefore, we assume a sediment thickness of 1000 and 1200 m, consistent with the respective ocean crust subsidence models[4, 5]. Assuming a sediment cover of ~200 m on top of the respective guyots[8], the total height of the guyots is ~4000-3800 m (guyot height of 3000 m + 1000 or 1200 m sediment - 200 m sediment on top). The normal subsidence of the oceanic crust (black solid line from [4] and black dashed line from [5]) indicates that the sampled structures have to be emplaced ~10-20 Ma after the ocean crust formation to form the flat tops by wave base erosion. Our age constraints yield a formation age of ~100-80 Ma. Note, if the guyots are covered by a thicker sediment cover, the formation ages are even older, whereas no sediments on top yield ~5 Ma younger age estimates. Source of bathymetric map (http://www.geomapapp.org[9]) and more details of a) are given in the Fig. 2 of the main article.

Supplementary References

1. Pearce JA. A user's guide to basalt discrimination diagrams. *Trace element geochemistry of volcanic rocks: applications for massive sulphide exploration Geological Association of Canada, Short Course Notes* **12**, 79-113 (1996).

2. Pearce JA. Geochemical fingerprinting of oceanic basalts with applications to ophiolite classification and the search for Archean oceanic crust. *Lithos* **100**, 14-48 (2008).

3. Geissler W*, et al.* The Rio Grande Rise and Jean Charcot Seamount Chain-microcontinents or the trail of the Tristan-Gough hotspot? Cruise No. MSM 82, 18 March 2019-24 April 2019, Montevideo (Uruguay)-Montevideo (Uruguay), RIOGRANDE. (2019).

4. Crosby A, McKenzie D, Sclater J. The relationship between depth, age and gravity in the oceans. *Geophysical Journal International* **166**, 553-573 (2006).

5. Stein CA, Stein S. A model for the global variation in oceanic depth and heat flow with lithospheric age. *Nature* **359**, 123-129 (1992).

6. Klingelhoefer F*, et al.* Imaging proto-oceanic crust off the Brazilian Continental Margin. *Geophysical Journal International* **200**, 471-488 (2014).

7. Perch-Nielsen K*, et al.* Site 356: Sao Paulo Plateau. (ed^(eds) (1977).

8. Caplan-Auerbach J, Duennebier F, Ito G. Origin of intraplate volcanoes from guyot heights and oceanic paleodepth. *Journal of Geophysical Research: Solid Earth* **105**, 2679-2697 (2000).

9. Ryan WBF*, et al.* Global Multi-Resolution Topography synthesis. *Geochemistry, Geophysics, Geosystems* **10**, (2009).
