# Supplementary material for: Evidence for compositionally distinct upper mantle plumelets since the early history of the Tristan-Gough hotspot
Source: Nat Commun. 2023 Jul 3;14:3908. doi: 10.1038/s41467-023-39585-0 (PMC10318034; doi:10.1038/s41467-023-39585-0)
Supplement: Supplementary file 3 — Description of Additional Supplementary Files [file 41467_2023_39585_MOESM3_ESM.pdf]

Description of Additional Supplementary Files

Supplementary Data: The file contains the measured Sr-Nd-Pb-Hf isotope ratios of the Rio Grande Rise and Jean Charcot Seamounts, initial and source corrected isotope ratios, as well as standard and sample replicates, and a data compilation of the Tristan-Gough hotspot track.
